# *OGDHL* Expression as a Prognostic Biomarker for Liver Cancer Patients

**DOI:** 10.1155/2019/9037131

**Published:** 2019-10-17

**Authors:** Yan Jiao, Yanqing Li, Zhuo Fu, Lin Hou, Qingmin Chen, Yujie Cai, Peiqiang Jiang, Miao He, Zhaoying Yang

**Affiliations:** ^1^Department of Hepatobiliary and Pancreatic Surgery, The First Hospital of Jilin University, Changchun, Jilin 130021, China; ^2^Department of Pathophysiology, College of Basic Medical Sciences, Jilin University, Changchun, Jilin 130021, China; ^3^Department of Hand and Foot Surgery, The First Hospital of Jilin University, Changchun, Jilin 130021, China; ^4^Cancer Center, The First Hospital of Jilin University, Changchun, Jilin 130021, China; ^5^Department of General Surgery, The Second Hospital of Jilin University, Changchun 130022, China; ^6^Department of Anesthesia, The Second Hospital of Jilin University, Changchun 130022, China; ^7^Department of Breast Surgery, China-Japan Union Hospital of Jilin University, 126 Xiantai Street, Changchun 130033, China

## Abstract

**Background and Objective:**

Liver cancer is a highly malignant tumor, and patients typically have poor prognoses. Metabolic reprogramming is a hallmark of cancer, and downregulation of oxoglutarate dehydrogenase-like (OGDHL) contributes to the onset and progression of several cancers. We examined the role of altered *OGDHL* expression in liver cancer and determined its value as a diagnostic and prognostic indicator for patients.

**Material and Methods:**

R (version 3.5.1) and several R extensions were used for data mining of The Cancer Genome Atlas (TCGA) dataset (including RNAseq and clinical information) and statistical analysis. Receiver operating characteristic analysis was used to determine the diagnostic value of *OGDHL*. The chi-squared test was used to identify the clinical correlates of *OGDHL* downregulation. Survival analysis (with the log-rank test) and univariate and multivariate Cox analysis were used to evaluate the effect of *OGDHL* expression on overall survival (OS) and relapse-free survival. TCGA was used for analysis of gene set enrichment.

**Results:**

*OGDHL* had lower expression in cancerous liver tissues than noncancerous adjacent tissues, and low expression correlated with more advanced patient age, histologic grade, stage, T classification, and poor survival. Patients with lower *OGDHL* expression had shorter OS and relapse-free survival. Multivariate Cox regression indicated that low *OGDHL* expression was an independent risk factor for poor prognosis. Gene set enrichment analysis indicated enrichment of the mitotic spindle, G2M checkpoint, and E2F targets in the *OGDHL* low expression phenotype.

**Conclusion:**

*OGDHL* has potential as a diagnostic and prognostic biomarker for liver cancer.

## 1. Introduction

Liver cancer is one of the most common digestive cancers in the world [1]. Although there have been improvements in clinical treatments in recent years, there have not been significant improvements in the prognosis of affected patients. There is an urgent need to identify novel prognostic biomarkers for liver cancer so that treatment selection can be improved.

Metabolic reprogramming is one of the hallmarks of cancer. Oxoglutarate dehydrogenase-like (*OGDHL*) is an essential regulatory gene and a putative tumor suppressor gene. The OGDHL protein is an isoform of 2-oxoglutarate dehydrogenase and functions as the first and rate-limiting step of the multienzyme OGDH complex (OGDHC), which degrades glucose and glutamate [2, 3]. Previous studies have reported enrichment of OGDHL in the brain and undetectable levels in the heart [2]. Subsequent studies examined the downregulation and methylation of *OGDHL* in breast cancer [4], cervical cancer [5], and colorectal cancer [6].

However, the diagnostic value, prognostic value, and role of OGDHL in liver cancer remain unknown. In this study, we compared *OGDHL* expression in cancerous and healthy liver tissues and evaluated its diagnostic value by receiver operating characteristic (ROC) analysis. We also examined the correlation of *OGDHL* expression with clinical features and performed survival analysis using the Cox model to assess its function as an independent prognostic indicator in liver cancer.

## 2. Materials and Methods

### 2.1. Data Mining of The Cancer Genome Atlas Database

The RNAseq data of *OGDHL* and clinical information were downloaded from The Cancer Genome Atlas (TCGA) dataset. No ethical approval was necessary because these are anonymized public datasets.

### 2.2. Statistical Analysis

All data analyses were performed using R (version 3.5.1) [7] and several R extensions. Boxplots were used to display expression of *OGDHL* mRNA. The chi-squared test was used to evaluate the correlation between *OGDHL* expression and the clinical features of patients. The pROC package was used to perform ROC analysis, to determine the optimal *OGDHL* cut-off point and to assess the diagnostic value of *OGDHL* expression by calculation of the area under the curve (AUC) [8]. Survival curves were plotted for different groups of patients, and curves were compared using the log-rank test. A survival package executed univariate and multivariate Cox analyses [9].

Ggplot2 was used for data visualization [10].

### 2.3. Gene Set Enrichment Analysis

Gene set enrichment analysis (GSEA) was used to assess the distributions of predefined gene sets in gene lists sorted by phenotype correlation and to determine the contribution of different genes to phenotype [11, 12]. This analysis was performed using the GSEA 3.0 software and the gene set of “h.all.v6.2.symbols.gmt” from the Molecular Signatures Database. The normalized enrichment score (NES) was obtained from 1000 permutations.

## 3. Results

### 3.1. Patient Characteristics and OGDHL Expression

**Figure 1:**
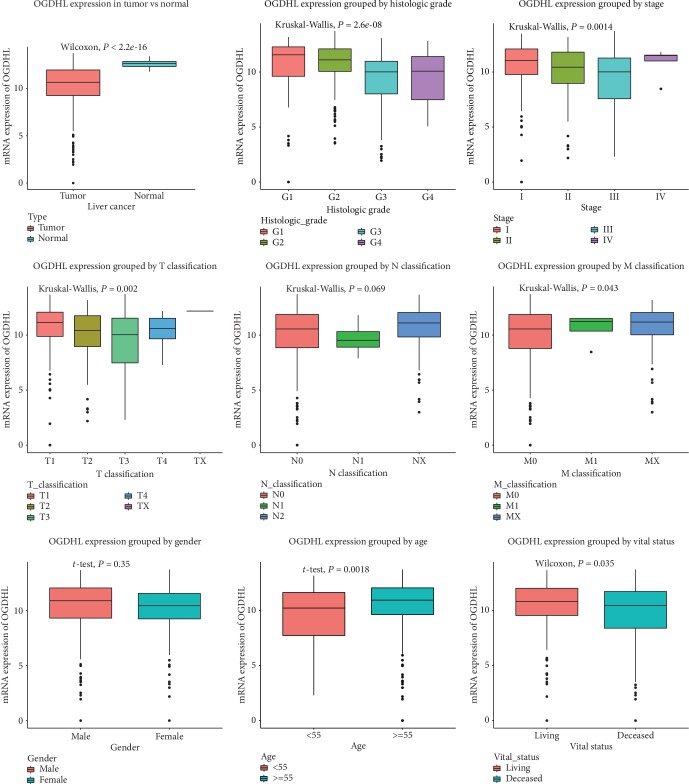
Expression of *OGDHL* in cancerous *vs*. normal liver tissues and in groups with different histologic grade, stage, TNM classification, sex, age, and vital status.

Table 1 shows the clinical characteristics of the 373 liver cancer patients from TCGA dataset, including age, sex, histological type, histologic grade, stage, TNM classification, receipt of radiation therapy, presence of residual tumor, vital status, and relapse. Analysis of *OGDHL* expression (Figure 1) indicated significantly lower expression in cancerous liver tissues than adjacent normal tissues (*P* < 2.2 × 10^−16^). In addition, *OGDHL* expression was inversely correlated with more advanced histologic grade (*P* = 2.6 × 10^−8^), stage (*P* = 0.0014), T classification (*P* = 0.002), M classification (*P* = 0.043), and age (*P* = 0.0016) but positively correlated with longer survival (*P* = 0.035).

### 3.2. Diagnostic Capability of OGDHL Expression and Correlation with Clinical Features

**Figure 2:**
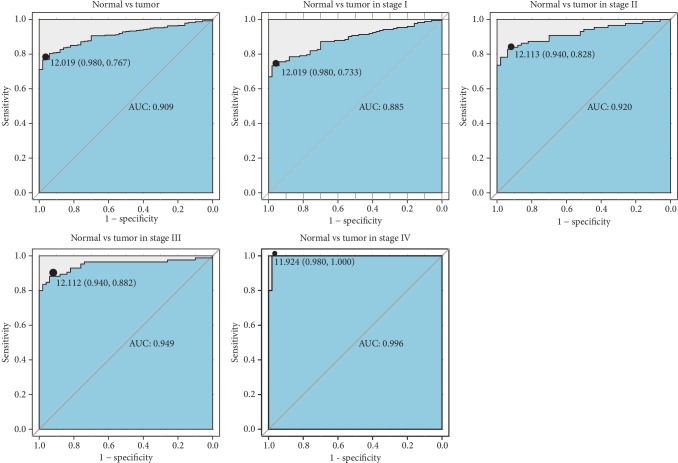
ROC analysis of the performance of *OGDHL* expression in identification of cancerous *vs*. normal tissues in all patients and subgroup analysis of patients with different stages of liver cancer.

We performed receiver operating characteristic (ROC) analysis to determine the diagnostic value of *OGDHL* expression (Figure 2). *OGDHL* expression had excellent diagnostic value overall (AUC = 0.909) and was also able to distinguish noncancerous tissue from stage I cancer (AUC = 0.885), stage II cancer (AUC = 0.920), stage III cancer (AUC = 0.949), and stage IV cancer (AUC = 0.998). We also found that low *OGDHL* expression correlated with more advanced patient age (*P* = 0.009), histologic grade (*P* = 0.000), stage (*P* = 0.015), T classification (*P* = 0.020), and poor survival (*P* = 0.037) (Table 2).

### 3.3. Correlation of OGDHL Expression with Survival

**Figure 3:**
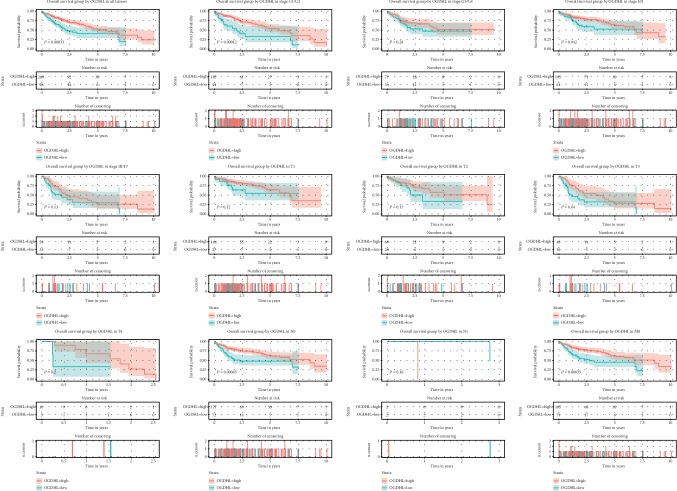
Relationship of *OGDHL* expression with OS in all patients and subgroup analysis of patients with different classifications of liver cancer (G1/G2, G3/G4, I/II, III/IV, T1-T4, N0, N1, and M0).

**Figure 4:**
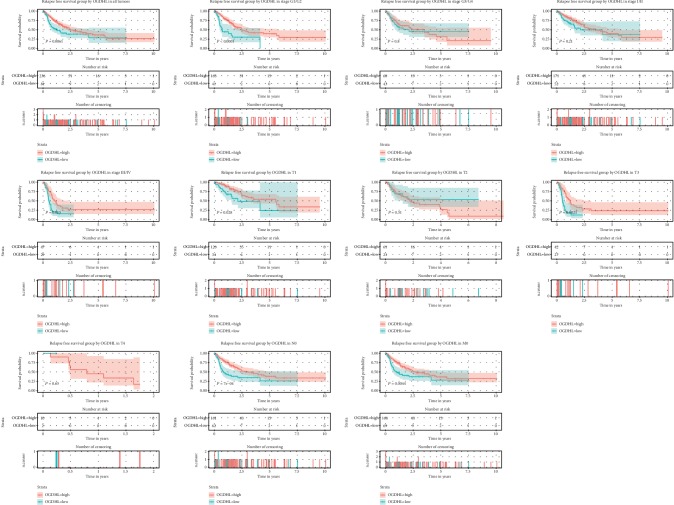
Relationship of *OGDHL* expression with relapse-free survival in all patients and subgroup analysis of patients with different classifications of liver cancer (G1/G2, G3/G4, I/II, III/IV, T1-T4, N0, and M0).

Survival analysis showed that patients with lower *OGDHL* levels had shorter overall survival (OS), and subgroup analysis indicated this relationship also held for patients with grade G1/G2, stage I/II, T3, N0, and M0 cancers (Figure 3). In addition, patients with lower *OGDHL* levels had shorter relapse-free survival, and subgroup analysis indicated this relationship also held for patients with grade G1/G2, stage III/IV, T1, T3, N0, and M1 cancers (Figure 4).

### 3.4. Low OGDHL as an Independent Risk Factor for Survival

We initially used univariate Cox analysis to select the potential variables for multivariable analysis (Tables 3 and 4). The subsequent multivariate Cox regression analysis indicated that low *OGDHL* expression was an independent risk factor for poor OS (hazard ratio (HR) = 1.75; 95% confidence interval (CI) = 1.2 to 2.54; *P* = 0.003) and poor relapse-free survival (HR = 1.58; 95%CI = 1.09 to 2.3; *P* = 0.016).

### 3.5. OGDHL-Related Signaling Pathways

**Figure 5:**
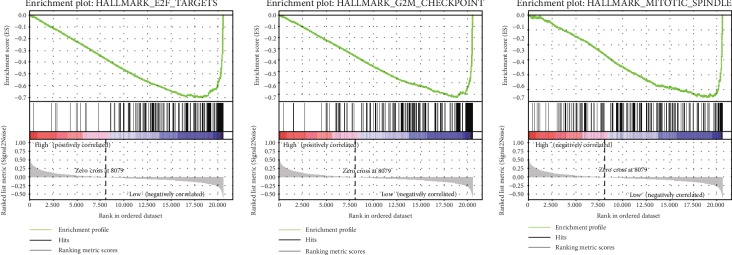
GSEA and identification of the potential targets of OGDHL as E2F, G2M checkpoint and mitotic spindle pathway.

We used GSEA to identify the signaling pathway(s) activated in HCC by comparing data sets that had low and high expression of *OGDHL* (Table 5, Figure 5). The results indicate significant differences in the enrichment of the MSigDB Collection (false discovery rate < 0.25, nominal *P* value < 0.05; h.all.v6.2.symbols.gmt). We then identified the most significant signaling pathways based on NES. These results show that E2F targets, the mitotic spindle, and the G2M checkpoint were enriched in the *OGDHL* low-expression phenotype.

## 4. Discussion

Our team previously used TCGA to identify diagnostic and prognostic biomarkers for several cancers [13–19]. In the present study, we found that *OGDHL* had low expression in liver cancer and that low expression correlated with more advanced patient age, histologic grade, stage, T classification, and shorter survival. In addition, our multivariable analysis indicated that low *OGDHL* expression was a significant diagnostic and prognostic biomarker for liver cancer.

Previous research identified OGDHL as an isoform of 2-oxoglutarate dehydrogenase, which regulated the degradation of glucose and glutamate [3]. An initial study of OGDHL function found enrichment of this protein in the brain but undetectable levels in the heart [2]. Subsequent studies focused on the relationship of *OGDHL* expression in several cancers and reported low expression in breast cancer [4], cervical cancer [5], and colorectal cancer [6]. Consistent with these results, we found low expression of *OGDHL* in liver cancer. Moreover, our ROC analysis showed that *OGDHL* expression had good diagnostic performance for patients with different stages of liver cancer, supporting its clinical use as a diagnostic biomarker. *OGDHL* expression also gradually decreased as histologic grade increased from G1 to G4, as stage increased from I to III, and as T classification increased from T1 to T3. The reason for the slightly higher expression in patients with the stage IV and T4 liver cancer is unknown, but it may be because we only analyzed a small number of patients with advanced cancer. We also found lower *OGDHL* expression in deceased than living patients, suggesting that OGDHL expression may be useful as a prognostic indicator.

Several previous studies have examined the functions of OGDHL. For example, Bunik and Degtyarev reported that OGDHL was located in the mitochondria (as predicted based on its sequence) and was an isoform of 2-oxoglutarate dehydrogenase [3]. Fujisawa et al. found that OGDHL functioned in adenylate kinase 4- (AK4-) regulated mitochondrial activity [20]. Yoon et al. identified nardilysin (NRD1) as a mitochondrial cochaperone for OGDH [21]. Sherrill et al. reported that certain variants of *OGDHL* lead to mitochondrial dysfunction and eosinophilic esophagitis [22]. Sen et al. found that OGDHL functioned as an antiproliferative gene and inhibited tumorigenesis *via* the AKT signaling pathway [5]. In conjunction with our results, this suggests that the downregulation of OGDHL, which alters mitochondrial function and increases cell proliferation, might explain our observation of a correlation of low *OGDHL* expression with more advanced cancer.

Many studies of *OGDHL* that examined its regulation of cancer have focused on methylation of its promoter region [4, 6, 23, 24]. However, no previous studies have examined its clinical significance or prognostic value. We found that patients with liver cancer who had lower *OGDHL* expression had shorter OS and shorter relapse-free survival. Our subgroup analysis indicated that *OGDHL* had prognostic value for specific groups of patients in predicting OS (G1/G2, I/II, T3, N0, and M0) and in specific group of patients for predicting relapse-free survival (G1/G2, III/IV, T1, T3, N0, and M1). These results suggest that *OGDHL* may be useful as a prognostic biomarker for liver cancer.

This study is the first to identify *OGDHL* as a novel diagnostic and prognostic biomarker for liver cancer. The targets of this protein appear to be the mitotic spindle, G2M checkpoint, and E2F. However, a limitation of this study is that we only examined a small number of patients with advanced-stage liver cancer; the cause of higher OGDHL expression in late stage liver cancer patients needs to be explored in the future study.

## 5. Conclusion

In conclusion, we found low expression of *OGDHL* in liver cancer and that low expression correlated with advanced patient age, histologic grade, stage, T classification, and poor survival. We also found that *OGDHL* expression had value as a diagnostic and prognostic indicator of liver cancer and that low *OGDHL* expression was an independent prognostic risk factor. Our GSEA analysis indicated that the potential targets of OGDHL were the mitotic spindle, G2M checkpoint, and E2F. This study is the first to identify the diagnostic and prognostic value of *OGDHL* in liver cancer, and our results indicate that *OGDHL* might be useful as a novel biomarker for liver cancer.

These results require verification by studies of larger populations.

## Figures and Tables

**Table 1:** Clinical characteristics of the liver cancer patients.

Characteristics	Number of pts (%)
Age	
<55	117 (31.45)
≥55	255 (68.55)
Gender	
Female	121 (32.44)
Male	252 (67.56)
Histological type	
Fibrolamellar carcinoma	3 (0.8)
Hepatocellular carcinoma	363 (97.32)
Hepatocholangiocarcinoma (mixed)	7 (1.88)
Histologic grade	
NA	5 (1.34)
G1	55 (14.75)
G2	178 (47.72)
G3	123 (32.98)
G4	12 (3.22)
Stage	
NA	24 (6.43)
I	172 (46.11)
II	87 (23.32)
III	85 (22.79)
IV	5 (1.34)
T classification	
NA	2 (0.54)
T1	182 (48.79)
T2	95 (25.47)
T3	80 (21.45)
T4	13 (3.49)
TX	1 (0.27)
N classification	
NA	1 (0.27)
N0	253 (67.83)
N1	4 (1.07)
NX	115 (30.83)
M classification	
M0	267 (71.58)
M1	4 (1.07)
MX	102 (27.35)
Radiation therapy	
NA	25 (6.7)
No	340 (91.15)
Yes	8 (2.14)
Residual tumor	
NA	7 (1.88)
R0	326 (87.4)
R1	17 (4.56)
R2	1 (0.27)
RX	22 (5.9)
Vital status	
Deceased	130 (34.85)
Living	243 (65.15)
Relapse	
No	179 (55.94)
Yes	141 (44.06)
OGDHL	
High	270 (72.39)
Low	103 (27.61)

**Table 2:** Relationship between the clinical features and OGDHL expression in liver cancer patients.

Clinical characteristics	Variable	No. of patients	OGDHL expression				*χ* ^2^	*P* value
			High	%	Low	%		
Age	<55	117	74	27.41	43	42.16	6.802	0.009
	≥55	255	196	72.59	59	57.84		

Gender	Female	121	86	31.85	35	33.98	0.072	0.788
	Male	252	184	68.15	68	66.02		

Histological type	Fibrolamellar carcinoma	3	3	1.11	0	0	1.809	0.617
	Hepatocellular carcinoma	363	261	96.67	102	99.03		
	Hepatocholangiocarcinoma (mixed)	7	6	2.22	1	0.97		

Histologic grade	G1	55	41	15.47	14	13.59	25.673	0.000
	G2	178	147	55.47	31	30.1		
	G3	123	69	26.04	54	52.43		
	G4	12	8	3.02	4	3.88		

Stage	I	172	135	54	37	37.37	10.116	0.015
	II	87	60	24	27	27.27		
	III	85	51	20.4	34	34.34		
	IV	5	4	1.6	1	1.01		

T classification	T1	182	144	53.73	38	36.89	10.765	0.020
	T2	95	64	23.88	31	30.1		
	T3	80	49	18.28	31	30.1		
	T4	13	10	3.73	3	2.91		
	TX	1	1	0.37	0	0		

N classification	N0	253	178	65.93	75	73.53	3.519	0.149
	N1	4	2	0.74	2	1.96		
	NX	115	90	33.33	25	24.51		

M classification	M0	267	186	68.89	81	78.64	3.523	0.156
	M1	4	3	1.11	1	0.97		
	MX	102	81	30	21	20.39		

Radiation therapy	No	340	245	97.22	95	98.96	0.320	0.572
	Yes	8	7	2.78	1	1.04		

Residual tumor	R0	326	239	90.53	87	85.29	4.018	0.245
	R1	17	12	4.55	5	4.9		
	R2	1	1	0.38	0	0		
	RX	22	12	4.55	10	9.8		

Vital status	Deceased	130	85	31.48	45	43.69	4.371	0.037
	Living	243	185	68.52	58	56.31		

**Table 3:** Univariate analysis and multivariate analysis of liver cancer patients' overall survival.

Parameters	Univariate analysis			Multivariate analysis		
	Hazard ratio	95% CI (lower~upper)	*P* value	Hazard ratio	95% CI (lower-upper)	*P* value
Age	1	0.69-1.45	0.997			
Gender	0.8	0.56-1.14	0.220			
Histological type	0.99	0.27-3.66	0.986			
Histologic grade	1.04	0.84-1.3	0.698			
Stage	1.38	1.15-1.66	0.001	0.83	0.67-1.04	0.105
T classification	1.66	1.39-1.99	0.000	1.84	1.46-2.32	0.000
N classification	0.73	0.51-1.05	0.086			
M classification	0.72	0.49-1.04	0.077			
Radiation therapy	0.51	0.26-1.03	0.060			
Residual tumor	1.42	1.13-1.8	0.003	1.38	1.08-1.77	0.011
OGDHL	1.93	1.34-2.79	0.000	1.75	1.2-2.54	0.003

**Table 4:** Univariate analysis and multivariate analysis of liver cancer patients' relapse-free survival.

Parameters	Univariate analysis			Multivariate analysis		
	Hazard ratio	95% CI (lower~upper)	*P* value	Hazard ratio	95% CI (lower-upper)	*P* value
Age	0.9	0.63-1.28	0.550			
Gender	0.99	0.7-1.41	0.966			
Histological type	2.02	0.66-6.24	0.220			
Histologic grade	0.98	0.8-1.21	0.883			
Stage	1.66	1.38-1.99	0.000	1.09	0.85-1.41	0.497
T classification	1.78	1.49-2.12	0.000	1.69	1.3-2.19	0.000
N classification	0.97	0.67-1.4	0.874			
M classification	1.17	0.79-1.74	0.432			
Radiation therapy	0.74	0.26-2.16	0.584			
Residual tumor	1.28	1.01-1.61	0.042	1.3	1.03-1.66	0.030
OGDHL	1.66	1.15-2.39	0.007	1.58	1.09-2.3	0.016

**Table 5:** Gene sets enriched in phenotype high.

NAME	ES	NES	NOM *P* value	FDR *q* value
HALLMARK_MITOTIC_SPINDLE	0.608	1.963	0.000	0.027
HALLMARK_G2M_CHECKPOINT	0.763	1.930	0.000	0.019
HALLMARK_E2F_TARGETS	0.748	1.881	0.002	0.020

Notes: gene sets with NOM *P* value < 0.05 and FDR *q* value < 0.25 are considered as significant. Abbreviations: FDR: false discovery rate; NES: normalized enrichment score; NOM: nominal.

## Data Availability

The raw data used in this study have been deposited in TCGA database (https://cancergenome.nih.gov/).
